# OpenEHR and General Data Protection Regulation: Evaluation of Principles and Requirements

**DOI:** 10.2196/medinform.9845

**Published:** 2019-03-25

**Authors:** Duarte Gonçalves-Ferreira, Mariana Sousa, Gustavo M Bacelar-Silva, Samuel Frade, Luís Filipe Antunes, Thomas Beale, Ricardo Cruz-Correia

**Affiliations:** 1 Center for Health Technology and Services Research Porto Portugal; 2 Healthy Systems Porto Portugal; 3 Ars Semantica London United Kingdom

**Keywords:** health information interoperability, electronic health record, data protection, GDPR, openEHR

## Abstract

**Background:**

Concerns about privacy and personal data protection resulted in reforms of the existing legislation in the European Union (EU). The General Data Protection Regulation (GDPR) aims to reform the existing directive on the topic of personal data protection of EU citizens with a strong emphasis on more control of the citizens over their data and in the establishment of rules for the processing of personal data. OpenEHR is a standard that embodies many principles of interoperable and secure software for electronic health records (EHRs) and has been advocated as the best approach for the development of hospital information systems.

**Objective:**

This study aimed to understand to what extent the openEHR standard can help in the compliance of EHR systems to the GDPR requirements.

**Methods:**

A list of requirements for an EHR to support GDPR compliance and also a list of the openEHR design principles were made. The requirements were categorized and compared with the principles by experts on openEHR and GDPR.

**Results:**

A total of 50 GDPR requirements and 8 openEHR design principles were identified. The openEHR principles conformed to 30% (15/50) of GDPR requirements. All the openEHR principles were aligned with GDPR requirements.

**Conclusions:**

This study showed that the openEHR principles conform well to GDPR, underlining the common wisdom that truly realizing security and privacy requires it to be built in from the start. By using an openEHR-based EHR, the institutions are closer to becoming compliant with GDPR while safeguarding the medical data.

## Introduction

The computer-based patient record has been considered an essential technology for health care in the last 25 years [1] even though their cost-effectiveness still needs more research to be fully assessed [2]. Currently, health care activities strongly rely on collected patient data and are feeding big data-driven health care projects that, among other aims, seek reliable predictors of health outcomes [3]. Health care professionals deal with a great volume of data, as their activities are heavily dependent on information accessed, as well as the way it is processed, managed, and made available.

Information technology (IT) development has enabled health care institutions to improve the collection and processing of health data, raising new concerns regarding the sensitivity of the information processed by information systems (ISs), namely, the risks concerning patient data protection and privacy. Although easy access to information is crucial to routine clinical practice, privacy, and security of medical information, it cannot be neglected, considering the consequences the misuse of medical information can present to the patient’s personal life. The Health Insurance Portability and Accountability Act privacy and security rules clearly emphasize the need of privacy of health information of the patient while allowing for sharing among different agencies [4].

One example of patient data misuse is the use of medical records for research without consent. This misuse is unfortunately widespread in the institutions that we have contact with. Often, this is a concern after the patient data are already accessed and just before the research is sent for publishing. This attracts little scrutiny compared with, for instance, biospecimen research, where concerns about genomic privacy prompted recent US federal proposals to mandate consent [5]. Moreover, cybersecurity threats against health care organizations are rising in numbers and severity [6,7]. New threats include hacktivism and organized crime, targeting individual identified data stored in hospitals and offices. New technologies and policies are needed to address the risks [8].

Health care data standards such as HL7 v2, HL7 FHIR [9], DICOM [10], or openEHR are central in the quality of the implemented patient records and have also tried to address security issues.Nevertheless, the pressure on health care to comply to new data protection rules is rising, so these standards must also be revisited taking this into consideration.

### General Data Protection Regulation

The European Union (EU) General Data Protection Regulation (GDPR) is the most important change in data privacy regulation in 20 years [11]. The GDPR replaces the Data Protection Directive 95/46/EC in an increasingly data-driven world that is vastly different from the time in which the 1995 directive was established. The GDPR was designed to harmonize data privacy laws across Europe to protect and empower all EU citizens’ data privacy and to reshape the way organizations across the region approach data privacy. Although the key principles of data privacy still hold true to the previous directive, many changes have been proposed to the regulatory policies. The GDPR was adopted on 27 April 2016. It becomes enforceable from 25 May 2018, after a 2-year transition period at which time those organizations in noncompliance will face heavy fines. So, it is of utmost importance for health care institutions to acknowledge the regulation’s requirements, analyze what is imposed in the obligations, and verify the compliance of the organization and its ISs, as well as, when necessary, to define strategies to adopt necessary measures.

Regarding principles relating to processing of personal data, the GDPR includes lawfulness and transparency toward the data subject; there must be a clear purpose for data collection and limitation regarding the further processing of data other than archiving; data processing should be adequate, relevant, and limited to what is necessary, fulfilling the principle of data minimization; personal data should be accurate and, when necessary, kept up-to-date (accuracy); personal data should be kept in a form, which permits identification of data subjects for no longer than is necessary (storage limitation); personal data should be processed in a manner that ensures its appropriate security (integrity and confidentiality); and the controller shall be responsible for and be able to demonstrate compliance with the regulation (accountability).

Regarding the rights of the data subjects, the GDPR defines the rights that controllers must make possible, such as the rights to be informed, of access by the data subject, to rectification, to erasure (commonly known as the *right to be forgotten*), to portability, to limitation to processing, to restriction of processing, to not be subject to automated individual decision making, including profiling, and to withdraw consent for processing of data. The controllers are obliged to implement technical and organizational measures that can answer to the data subject’s request, regarding their rights, but also at a security level.

Organizations must incorporate concepts such as privacy by design and by default in the development of their systems, to comply with GDPR requirements related to the protection of the personal data they process. Even though these requirements are seen as a restriction for medical research, there are pointers in the literature to the standardization of the data in Europe and uniformization of a digital single market together with the GDPR, which will facilitate medical research when the research is considered in the public interest [12].

### OpenEHR

OpenEHR presents a set of principles for an interoperable EHR systems architecture based on a multilevel, single source modeling approach. OpenEHR’s specifications are published by the openEHR Foundation, an entity responsible for the development of the specifications and the availability of specific tools enabling the standard’s use. One of the main goals of openEHR is to enable the development of EHR systems to be able to communicate with each other, without loss of meaning, thus achieving semantic interoperability.

Modeling in openEHR relies on a 2-level scheme that separates the content from the form in which it is defined. The openEHR Architecture Overview states that, under the 2-level approach, the first level is a stable reference information model, which defines basic concepts for logical data representation, which also act as primitives for the second layer of models. These primitives include data types, structures, and the connections between them controlling how they can be assembled to create clinical content definitions in the second level. The clinical content definitions consist of data points, and groups are defined in the form of constraint structures, known as *archetypes*, on the first layer primitives. Archetypes can be used to create other more complex archetypes and also *templates* which are representations of datasets for specific domain use cases. Thus, an openEHR Archetype is the model (or pattern) for the capture of clinical information—machine-readable specification of how to store patient data using the openEHR Reference Model whereas archetypes describe complete domain-level data structures such as *diagnosis* or *test result*, and a template provides the means for grouping archetype-defined data points for particular business purposes.

With this 2-level approach, the clinical content is structured *outside the software*, allowing EHR systems to be more flexible, as the modifications concerning the clinical knowledge are realized solely by the modifications of the archetypes, without compromising the integrity of the software or data of an EHR repository which is based on the Reference Model [13].

OpenEHR offers a new paradigm of systems modeling, relying on a very stable model at the software level and a very flexible modeling that reflects the evolution of knowledge at the domain level. There are tools to help the modeling process such as the Clinical Knowledge Manager which is a Web-based repository that contains archetypes and templates developed by an international group of specialists. This platform supports collaboration open to everyone (specially clinicians, IT professionals, and software engineers), where participants can author, review, translate, and maintain archetypes and templates.

### Aim

Given the nature of openEHR as a standard being used to build EHR systems, it is important to understand to what extent the openEHR principles address the requirements mandatory to GDPR. This research aimed to study if and how openEHR addresses the GDPR requirements.

## Methods

The study was performed in 3 steps: (1) identify the requirements for a health information system (HIS) compliant with GDPR; (2) identify the openEHR security principles regarding the functionalities of an HIS; and (3) determine the correspondence of the openEHR principles to the GDPR requirements.

### General Data Protection Regulation Requirements

The list of the GDPR requirements was created by reading the legislation by specialists (authors of this paper: LFA and MS). The list of requirements was built with a strong input on the global description of the system, focusing on the identification of the GDPR goals and the later translation to system functionalities. The requirements were described using the Institute of Electrical and Electronics Engineers Guide for Developing System Requirements Specifications [14].

A search was conducted on the PubMed database for papers related to the GDPR using the GDPR keyword. The search returned a list of 29 papers from which the ones without an abstract as well as the ones that did not relate specifically to the subject in hand were removed. We were left with 5 papers that were reviewed to obtain higher level groups for the GDPR requirements.

### OpenEHR Principles

The list of openEHR architectural features relevant to GDPR was compiled from the openEHR Architecture Overview [13] by specialists (authors of this paper: GB and SF), aiming to identify its main principles in view of the functionalities of an openEHR-based system. A description of each principle was agreed upon by the specialists.

We identified and listed the openEHR features, with a strong focus on the functionalities of a system rather than the implications of the architecture.

### Matching General Data Protection Regulation Requirements With OpenEHR Features

Each feature can match more than one requirement, and a requirement can be matched by more than one feature. To be considered as a match, the openEHR features should meet the GDPR requirements in a straightforward way by the simple implementation of its architecture.

## Results

The results section presents the (1) list of the GDPR requirements, (2) the openEHR GDPR–related features, (3) a table that matches the requirements with the features, and (4) a list of requirements not met by openEHR GDPR–related features.

### General Data Protection Regulation Requirements

The article review on the GDPR in PubMed left us with a list of 5 articles that were relevant for the subject of GDPR in health care. Moreover, 2 of the 5 articles focus on data sharing [15,16]and set Consent, Privacy, Security Measures, Adequacy of use, and Oversight as a high-level grouping of GDPR concerns in health care data sharing. Furthermore, another 2 articles were related to the concerns of the GDPR in research [17] and in the area of radiology [10]. Although the first focus was on a review of the GDPR for medical research, it does not provide a usable division of the requirements, focusing instead on the changes GDPR brings to researchers; the second focus was on term definitions such as portability of health care data, personal data breaches, anonymization, pseudonymization, and encryption, in which requirements affect the lawful processing of data for research. The final article describes a system that focuses on the audit and traceability helping institutions fulfill the need that GDPR imposes in the institution to know who, when, and what is done with their data [18]. On top of these articles, we analyzed an article by Mense and Blobel [9] where the authors analyze the GDPR requirements, extract some key factors from the legislation, and match them against multiple HL7 standards including CDA, FHIR, and HL7 v2. They synthetize the GDPR legislation into 7 key factors that we enumerate next:

1. Data protection by design and by default.
2. Data portability.
3. Right to be forgotten—notification requirement.
4. Unambiguous consent.
5. Privacy notices.
6. Right to Access and Records of processing activities.
7. Explicit and formally represented policies.

In our analyses of the GDPR legislation [11], we identified a total of 50 requirements grouped into 7 groups. The requirements were aggregated into the following groups:

1. Limitations to data processing, which include requirements directly related with data processing limitation for the institution.
2. Data quality and accountability, which include requirements related with integrity, accuracy, and audit.
3. Consent by data subject, requirements related with consent and authorization.
4. Empowerment of data subject, requirements that increase the rights of the data subjects on their data.
5. Data breaches, requirements directly related with data breaches and how to proceed in case of a data breach.
6. Data portability and interoperability, requirements related to authorized data sharing.
7. Privacy control and impact assessment, requirements related with Privacy Impact Assessment (PIA) and privacy by default and by design.

The complete list of the requirements is defined in Textbox 1.

### OpenEHR Design Principles

The following 8 features were identified in openEHR as being relevant to GDPR:

#### Feature 1: 2-Level Modeling

2-level modeling promotes the separation of the reference model (stable information model that defines the logic structure of the EHR and the demographic data) from the content model (the definition of clinical domain as archetypes and templates modeled by clinical professionals. Essentially, archetypes and templates are datasets external to any system’s software). The Reference Model is implemented at the software level, whereas the Domain Model is set through the archetype and template modeling. This results in the separation and independence of software structure from its content, enabling flexible, interoperable, and scalable health systems. Fundamentally, all openEHR systems support the same data structure and remain able to communicate, regardless of how many changes are made to domain information definitions.

#### Feature 2: Separation of Clinical and Demographic Information

One of the openEHR design principles is to enable the complete separation of the EHR from identifiable demographic information via separated repositories with flexible referencing. In case of a data breach of the EHR repository, it allows the identity of the data subject to be preserved, unless the demographic repository is also breached. This principle strengthens the data subject’s anonymization regarding the information in their EHR, as it is used as an instance in the EHR, called *Party_Self*, to make a reference to the data subject. This information works as an optional external reference, such that the EHR can be set to provide 3 levels of separation. The external identifier is determined in the instance *Party_Self* by

- Nowhere in the EHR (every *Party_Self* instance is left empty). This is the safest way, and it means that the connection between the EHR and the patient needs to be made outside the EHR, by connecting the EHR identifier (EHR.ehr_id) and the subject’s identifier.
- Only once in the *EHR_STATUS* object (subject’s attribute) and nowhere else. It is a very safe measure if the EHR_STATUS object is protected in any way.
- In any *Party_Self* instance, this is a reasonable solution in a safe environment appropriate to copy parts of the record on demand.

#### Feature 3: Service Model

openEHR service model [19,20] specifies a formal, abstract definition of interfaces to be implemented in an openEHR system. Implementations can follow these abstract definitions to allow interoperability between various implementations. The service model currently consists of the EHR Service, the Query Service, and the Definitions Service. The EHR Service allows the consultation of data made available by the EHR Application Programming Interface (API). The detail level of the consulted data may vary, allowing the access to more complex records, such as changes in versions, or it may allow the search of simpler elements, such as single clinical data items, patient identifications, etc. The Definitions Service allows access to an archetype repository, acting as an important tool for access to important information by medical professionals (eg, if they need an archetype that is not on his/her local repository for certain medical treatment). The service model thus takes on an important role in controlling the availability of data, as well as the possibility of consultation, allowing the definition of safe and intuitive views.

#### Features 4 and 5: Version Control—Versioning and Digital Signature

Important openEHR features related to GDPR relate to data integrity support. The EHR or demographic repository is managed using *Versioned Objects*. Versioned Objects are used to contain the versions of a *Composition* or *Party* structure, which in turn contain fine-grained clinical and demographic data, respectively. The set of changes to items in any update to the system is called a *Contribution* (more commonly known as a *change set*). Each change set works as a transaction, ensuring the consistency and integrity of the data repository. Changes made by users (creating new records, deleting records, modifying records, and transferring records) are not performed at the item/record level but at the level of the repository as a whole. This means that no version is deleted or modified; all the changes are physically implemented as new versions in the repository. This principle ensures indelibility (no information is deleted). Version control includes the possibility of each version having a digital signature, created as a primary-key encrypted of a hash of a representation approved of the compromised version. In a versioned system, the digital signature acts as a verification of integrity, a measure of authentication and also as a measure of nonrepudiation.

**List of General Data Protection Regulation requirements.**

1. Limitations to data processing
  - Purpose limitation: The system shall admit the definition of a purpose for the limitation of processing.
  - Data minimization: The system must allow the definition of the minimum of data fields required for processing.
  - Period of storage limitation: The system must allow the definition of deadlines for the processing of specific personal data, in order with the purpose of processing.
  - Method storage limitation: The system must allow the storage of data in a way that only identifies the data subjects during the necessary time relative to the purpose.
  - Limitation of processing of personal data: The system must be able to limit the processing of personal data according to the consent given by the data subject.
2. Data quality and accountability
  - Accuracy: The system must allow the update of the personal data whenever necessary.
  - Integrity and confidentiality: The system must support the adoption of technical and organizational measures that ensure the security of processing, namely, the protection against unauthorized processing or against the loss, destruction, or accidental harm of personal data.
  - Accountability: The system must allow the demonstration of compliance with the data processing principles.
  - Statement of accountability: The system must support the demonstration of compliance with codes of conduct and certified procedures.
  - Conditions of processing: The system must record data describing the legal context that allows the processing of data.
  - Record of processing: The system shall be able to keep an up-to-date and accurate record of all the processing activities and must allow the record of processing to be written in an electronic format.
  - Availability of records of processing: The system must allow access to consult its records of processing.
  - Location of data: The system must be able to identify and locate a subject’s data that must be limited inside the system.
3. Consent by data subject
  - Explicit consent: The system must be able to record and show the consent of the data subjects for the collection of personal data and the purpose for collecting.
  - Management of consent: The system must allow changes to the consent by the data subject.
  - Record of consent: The system must be able to keep a record of consent or consents to distinguish it from other content.
  - Withdrawal of consent: The system must ensure the ability to withdraw consent (opt-out) in an easy and clear way, using the same means in which the consent was obtained.
  - Features of the consent: The system must ensure that the consent provision by a subject is active, not obtained through silence, inactivity, or prechecked boxes, and that it is confirmed in words.
  - Lawfulness of processing after withdrawal of consent: The system shall be able to ensure the lawfulness of data processing after the withdrawal of consent.
  - Objection to processing: The system must support the cessation of processing in response to a request by the data subject.
4. Data subject empowerment
  - Information provided to data subject: The system must inform the data subject about the conditions and rules relating to data processing and privacy.
  - Means to provide information to data subjects: The system must have a means of providing such information.
  - Verification of the identity of the data subject: The system must allow the verification of the identity of data subjects upon the request.
  - Data subject request: The system must support the receipt of data subject’s request.
  - Response to request: The system must enable the solicitation to the data subject’s request by the same means the request was made.
  - Data subject access: The system must provide a copy of the data subject’s personal data at processing on request.
  - Data subject request action: The systems must support the means for the data subject to request access to the subject’s data.
  - Information accessed by the data subject—The system must enable access to the data subject’s information and actions such as the following:
    - Purpose of processing.
    - Categories of personal data held by the system.
    - The recipients or categories of recipients to whom personal data have been or will be disclosed.
    - The period for which personal data will be stored.
    - The existence of the right to request from the controller rectification or erasure or restriction of data processing.
    - Right to lodge a complaint with a supervisory authority.
    - Available information as to the source of data collection, if personal data were not collected from the data subject.
    - Existence of automated decision making, including profiling.
  - Response to data subject request: The system must allow the response to the data subject’s request in a commonly used means.
  - Data subject direct access: The system must provide a secure method for the direct access of the data subject to their personal data.
  - Personal data rectification: The system must allow the rectification of inaccurate personal data by the data subject.
  - Personal data erasure: The system must allow the erasure of personal data when consent is removed or when the purpose on which the data were gathered is no longer valid.
  - Legitimate interest: The system must be able to demonstrate to the data subject the legitimate interest for the processing, including retrieval, modification, and sharing.
  - Confirmation of data processing: The system must be able to confirm the processing of the data subject’s personal data in each case requested by the subject.
5. Data breaches
  - Records of data breaches: The system must keep a record of data breaches that were detected.
  - Records of data breaches nature: The system must register information regarding the nature of data breach.
  - Data breach description: The system must keep a record of information regarding the nature of data breach in a format subject to be sent to the supervisory authority.
  - Data breach notification deadline: The system must enable the notification procedure of the supervisory authority in 72 hours.
  - Data breaches notification procedures: The system must support the development of procedures for the report of internal breaches.
6. Data portability and interoperability
  - Portability of personal data: The system must allow the portability of personal data in a structured, common, automatic format.
  - Portability of personal data between controllers: The system must be able to transfer personal data to another controller.
  - Interoperability of systems and formats: The system must enable interoperability for the transfer and portability of personal data.
  - Communication between institutions: The system must allow the communication between institutions involved in the processing of the same personal data.
  - Cross-border data transfers: The system must allow the transfer of personal data to other countries.
  - Cross-border data transfers guarantee: The system must enable the recording of the proper measures presented by the third country or international organization that allows the transfer of personal data.
7. Privacy control and impact assessment
  - Privacy by design: The system must allow the pseudonymization and encryption of data and must be able to apply data minimization measures, storing only minimal needed data.
  - Privacy by default: The system must ensure the processing of personal data relevant to the purpose and it must ensure that no personal data are made available without human intervention.
  - Access control measures: The system must make data unintelligible in case of unauthorized access.
  - Data Protection Impact Assessment (DPIA) records preservation: The system must allow the preservation of DPIA.
  - DPIA consultation: The system must allow the consultation of the DPIA when the controller requires it.

#### Features 6 and 7: Access Control—Access Control List and Configurations

openEHR access control is set through the object named *EHR_ACCESS*. This object works as a gate to all access information, being that any decision regarding access information should be based on the policies and rules established in it. OpenEHR’s EHR allows the definition of an access control list, indicating the identified individuals and their categories. The definition of the access control list should consider relevance of the user’s identity access either in terms of time or duration of access. When creating an EHR, openEHR allows the definition of a gatekeeper responsible for the access control configurations. The gatekeeper becomes an identity recognized in the EHR, usually being the own patient (in case of mentally competent adults) or a relative or legal tutor (in case of being a child or mentally incapable). The gatekeeper sets who can make changes in the access control list, being that all changes are kept in the audit trail. These features could make use of recent developments in security technologies referred to in the literature [21,22], although they still need field validation in the health care area.

#### Feature 8: Audit Trailing

All changes that are made, at all levels, in the EHR are recorded in the audit trail, with data related to the identity of the user, timestamp, purpose (of the alterations performed), digital signature, and relevant version information.

### Matching General Data Protection Regulation Requirements With OpenEHR Principles

Table 1 presents the existing matches between the GDPR requirements and the openEHR principles.

The results obtained showed that openEHR GDPR–related features satisfied at least 1 identified GDPR requirement.

The GDPR requirement Method Storage Limitation (1.4), listed in Textbox 1, is fulfilled by the Separation of EHR and demographic information, allowing a separate storage of demographic and clinical data. The identity of the data subject is automatically preserved when the clinical and demographic information are separated. In that way, although the clinical data are stored for treatment, the demographic data are only connected to the EHR through an external identifier, allowing the identification of the data subject only during the necessary period of the purpose of processing (typically only on a device used by an authorized health professional during a health care process).

Integrity and Confidentiality (2.2), listed in Textbox 1, is a complex requirement in the GDPR and is thus fulfilled by a group of openEHR architectural features. Data versioning allows the creation of new EHR versions, ensuring indelibility, and is thus an important measure against the loss, destruction, or accidental corruption of EHR data, guaranteeing trustworthy and reliable information at all moments of processing. The digital signing of data ensures the authentication, nonrepudiation, and integrity of the EHR, acting as an important security and integrity measure of the personal data and its processing. The access control rules are included in the openEHR architecture by design, and it ensures the confidentiality of the patient’s data by limiting the occurrence of an unauthorized or illicit processing, because of the definition of who is authorized to access the data. There is also a component that identifies who can access or change the access control rules, it sets the individuals who can change the configurations of the access list, contributing to legitimate and justified accesses and ensuring the integrity of processing. On top of these preventive features, the openEHR services architecture [23] includes a System Log service that records access actions, as well as identity of the users, date and time, and justification of the action, ensuring the integrity of the data and postaccess analyses. (This service is not defined by openEHR but assumed to be an implementation of, eg, IHE ATNA.) These design features ensure the security of processing, correctly identifying when, who, what, and how data were accessed, and allowing a postaccess audit.

Accountability (2.3), Record of processing (2.6), and Availability of records of processing (2.7), listed in Textbox 1, are fulfilled by the audit trail feature, which allows the system to keep a record of all information related to the processing of data. This way, it is possible to demonstrate compliance with the principles and obligation of the requirements, as it is possible to see information related to access and actions taken in the EHR. Due to its traceability, it allows the creation of a record of personal data processing that can become available to the data authorities.

The requirement Verification of the identity of the data subjects (4.3), listed in Textbox 1, is fulfilled by the separation of demographic and clinical data that ensures the separation of identifiable information from the clinical data. The EHR has an identifier associated to a single patient, ensuring the identification of the data subject’s identity if necessary.

Data subject access (4.6) and Data subject direct access (4.10), listed in Textbox 1, are two of the requirements that we identified for the GDPR and can be fulfilled by the Access control feature of the openEHR. The data subject can be granted access using the access control list and can manage this list through the Access control configurations. Data subject direct access is fulfilled by an extra feature, the Service Model feature that allows the creation of direct access reference to the subject’s data by the subject.

Confirmation of data processing (4.14), listed in Textbox 1, requirement is fulfilled by the audit trailing feature of the openEHR principles. In the audit trail, systems can store every action that is done on the subject’s data. Also, it is possible to check what changes were done to the data through the different versions created in each action.

Data portability and interoperability are, by design, a key focus of the openEHR architecture and 2-level modeling, so the GDPR requirements 6.1, 6.2, 6.3, and 6.5 can be matched by these features. The interoperability point of the requirement 6.3 is also fulfilled with the integration of another of the openEHR principles, the Service Model that allows the creation of different interfaces, in different systems around the institution, using the same data. By supporting different views that allow the consultation of the same EHR, the record maintains its integrity and structure, ensuring interoperability.

Privacy by design and by default were identified as requirements 7.1 and 7.2 in Textbox 1. The separation of demographic and clinical data by design improves the protection of the data subject identification by separating the EHR from the identifiable demographic information, only relating them by an external identifier. By default, during health care, only the EHR data should be considered. Privacy by default is also matched by the Access control list and its configuration that endures the access and availability of information and ensures that the data are only processed and accessed for the purpose settled, by those authorized to do it, safeguarding the patient’s data privacy.

**Table 1:** List of the 17 General Data Protection Regulation (GDPR) requirements that are met by openEHR principles.

GDPR requirements	openEHR principles							
	2-level modeling	Separation of EHR and demographic information	Service model	Version control—versioning	Version control—digital signature	Access control—access control list	Access control—configurations	Audit trailing
Method storage limitation	—^a^	X^b^	—	—	—	—	—	—
Integrity and confidentiality	—	—	—	X	X	X	X	X
Accountability	—	—	—	—	—	—	—	X
Record of processing	—	—	—	—	—	—	—	X
Availability of records of processing	—	—	—	—	—	—	—	X
Verification of the identity of the data subjects	—	X	—	—	—	—	—	—
Data subject access	—	—	—	—	—	X	X	—
Data subject direct access	—	—	X	—	—	X	X	—
Confirmation of data processing	—	—	—	X	—	—	—	X
Portability of personal data	X	—	—	—	—	—	—	—
Portability of personal data between controllers	X	—	—	—	—	—	—	—
Interoperability of systems and formats	X	—	X	—	—	—	—	—
Cross-border data transfers	X	—	—	—	—	—	—	—
Privacy by design	—	X	—	—	—	—	—	—
Privacy by default	—	X	—	—	X	X	X	—

^a^Represents no match.

^b^X represents a match in the table.

### General Data Protection Regulation Requirements Not Met by OpenEHR Principles

Textbox 2 presents the 35 GDPR requirements that are not met by openEHR principles.

Regarding requirements from group 3, although none of the openEHR principles identified could match this requirement, the implementation of openEHR architecture could help to fulfill the requirement related to explicit consent through the creation of an archetype *Consent*. This archetype would allow the recording and management of Consent, allowing the controller to keep all the information necessary.

Regarding requirement 4.13, the information regarding the legitimate interest of processing could be included in an archetype, although even without this, the system can often infer legitimate access by analyzing, for example, hospital admission and discharge dates and association of subject to the general practitioner’s clinic. Regardless, better methods are needed in the future. In case of consent being the legitimate interest for the processing, this information could be recorded along with the consent.

**Requirements that are not met by openEHR principles. DPIA: Data Protection Impact Assessment.**

1. Limitations to data processing
  - Purpose limitation
  - Data minimization
  - Period of storage limitation
  - Limitation of processing of personal data
2. Data quality and accountability
  - Accuracy
  - Statement of accountability
  - Conditions of processing
  - Location of data
3. Consent by data subject
  - Explicit consent
  - Management of consent
  - Record of consent
  - Withdrawal of consent
  - Features of the consent
  - Lawfulness of processing after withdrawal of consent
  - Objection to processing
4. Data Subject empowerment
  - Information provided to data subject
  - Means to provide information to data subjects
  - Data subject request
  - Answer to request
  - Data subject request form
  - Information accessed by the data subject
  - Response to data subject request
  - Personal data rectification
  - Personal data erasure
  - Legitimate interest
5. Data breaches
  - Records of data breaches
  - Records of data breaches nature
  - Data breach description
  - Data breach notification deadline
  - Data breaches notification procedures
6. Data portability and interoperability
  - Communication between institutions
  - Cross border data transfers guarantee
7. Privacy control and impact assessment
  - Access control measures
  - DPIA records preservation
  - DPIA consultation

## Discussion

### Principal Findings

OpenEHR acts mainly on requirements that either shape the functional layer of the system or relate to data traceability, integrity, and confidentiality. Data protection by design, portability, and interoperability are ensured by openEHR's architecture because of the 2-level modeling and separation of clinical and demographic data. Personal data integrity and confidentiality are mainly addressed by the access control, versioning, and audit trail features.

Nevertheless, openEHR is a valuable tool for the fulfillment of requirements that are not directly met, such as definition of notification forms (for data subjects and data authorities), the creation of a means of communication for records, and preservation of Data Protection Impact Assessment and records of compliance with codes of conduct and certifications.

These requirements need to be addressed from an organizational point of view, either through the reform of existing processes or the definition of new ones. However, the versioning and audit trail features can support the recording of important information related to the data processing and data breaches.

Apart from the clinical content models (archetypes and templates), openEHR does not support the automatic creation of other needed documentation for GDPR such as a structure to store the consent but can be backed up by the principles (namely the traceability of the data, actions, and users).

OpenEHR’s architectural features can still help fulfill requirements related to consent. Versioning and audit trailing allow systems to record and verify any action or access made in the EHR. By acknowledging the deadline for the processing, it is possible to identify if there are any data being processed without the consent of the data subjects. Thus, even if the openEHR architecture does not include dedicated features for some GDPR requirements, it presents itself as an important support in relation to the processes that an institution must implement.

It should be noted that some of the GDPR requirements, namely the ones related to the organizational processes, are probably not satisfiable by any EHR architecture. However, it is important to note the organizational reforms that must be conducted require actions not only at the level of their organizational processes and services but also specifically at the level of their systems.

### Limitations

To our knowledge, there is no formal list of GDPR requirements for an EHR system. The list of requirements we propose in this study is intended as a starting point for further discussion and future work.

### Conclusions

OpenEHR is a promising approach to the development of EHR systems compliant with GDPR, allowing institutions to respond to functional needs focused on the privacy and security of health data. It is also a strong solution for issues related to data portability and data protection by default, which are now required by the regulation.

Primarily, openEHR is a good solution for issues related to privacy and data protection, the main goals of GDPR.

The use of IT has become essential to health care delivery. OpenEHR defines an integrated environment, focused on the provision of health care and access to quality information, which helps institutions conform to the GDPR requirements ensuring the privacy and protection of personal data.

## Abbreviations

EHR: electronic health record
DPIA: Data Protection Impact Assessment
EU: European Union
GDPR: General Data Protection Regulation
HIS: hospital information system
IS: information system
IT: information technology
